# The effects of small-quantity lipid-based nutrient supplements on adolescent physical activity and sedentary behaviour: a follow-up of the International Lipid-Based Nutrient Supplements-DYAD-Ghana trial

**DOI:** 10.1017/S0007114525105126

**Published:** 2025-09-28

**Authors:** Jonnatan Fajardo, Charles D. Arnold, Mavis O. Mensah, Ebenezer Adjetey, Maku E. Demuyakor, Xiuping Tan, Brietta M. Oaks, Seth Adu-Afarwuah, Amanda E. Guyer, Kathryn G. Dewey, Paul D. Hastings, Elizabeth L. Prado, Christine P. Stewart

**Affiliations:** 1 Department of Nutrition, Institute for Global Nutrition, University of California, Davis, CA, USA; 2 Department of Nutrition and Food Science, University of Ghana, Accra, Ghana; 3 McKing Consulting Corporation, Atlanta, GA, USA; 4 Department of Nutrition, University of Rhode Island, Kingston, RI, USA; 5 Department of Human Ecology and Center for Mind and Brain, University of California, Davis, CA, USA; 6 Department of Psychology and Center for Mind and Brain, University of California, Davis, CA, USA

**Keywords:** Lipid-based nutrient supplements, Adolescent, Physical activity, Sedentary behavior, Ghana

## Abstract

Few studies have examined the effects of early-life nutrition interventions on adolescent physical activity (PA). We aimed to examine the long-term effects of small-quantity lipid-based nutrient supplements (SQ-LNS) on adolescent PA and sedentary behaviour (SB) and to describe current adolescent PA and SB levels in this cohort. In the International Lipid-Based Nutrient Supplements (iLiNS)-DYAD-Ghana trial, 1320 mothers were enrolled and randomly assigned to one of three conditions: (1) daily iron and folic acid during pregnancy and placebo (calcium) from birth to 6 months postpartum (IFA), (2) multiple micronutrient supplements during pregnancy to 6 months postpartum (MMN) or (3) SQ-LNS during pregnancy to 6 months postpartum (LNS). Infants from mothers in the LNS group received SQ-LNS designed for children from 6 to 18 months. We recruited 11–13-year-old adolescents of mothers enrolled in the iLiNS-DYAD-G trial for a 7-d PA and SB assessment using accelerometers (*n* 305) and self-reported PA and SB (*n* 508). We compared the LNS with non-LNS (IFA+MMN) groups using ANCOVA models for the following outcomes: mean vector magnitude counts per minute, PAQ-C score and percentage of time in SB, light PA and moderate-to-vigorous PA (MVPA). There were no significant differences between the LNS and non-LNS groups in any PA outcome in minimally or fully adjusted models. Only approximately 50 % of adolescents met the PA recommendation of 60 min/d MVPA, with males more active than females; however, there is room for improvement. SQ-LNS in early life does not appear to have a sustained impact on PA or SB.

On a global scale, physical activity (PA) has decreased, as more than 25 % of all adults have become insufficiently active^(1)^. The rates of PA among adolescents are alarmingly low: the WHO estimates that over 80 % of adolescents worldwide do not meet the recommended 60 min/d of moderate-to-vigorous physical activity (MVPA)^(2)^. These rates of PA are concerning, given that sufficient PA offers substantial health benefits to adolescents, such as enhancing cardiovascular health, increasing bone mineral density and reducing the risk of non-communicable diseases^(3–5)^.

Despite the known benefits of PA for overall health, there is limited research investigating the effects of early-life nutrition on childhood PA patterns. Previous studies involving children from infancy to early childhood who received early-life nutritional supplementation, such as multiple micronutrient supplements and macronutrients or zinc, suggested positive effects on PA and motor activity compared with groups that did not receive nutritional supplementation^(6–8)^. However, studies examining the long-term effects of nutritional supplementation on PA into adolescence are scarce. One study, the Institute of Nutrition of Central America and Panama longitudinal study in Guatemala, examined the effect of a nutritional supplement on physical work capacity in adolescents and young adults^(9,10)^. The investigators found that males exposed to the micronutrient-fortified, high-protein ‘atole’ before 7 years of age had a higher maximal oxygen consumption than males who received the control ‘fresco’, a sweet fruit-flavoured drink. Similarly, females 11–14 years of age who were exposed to atole had a higher maximal oxygen consumption than females exposed to fresco.

Small-quantity lipid-based nutrient supplements (SQ-LNS) may offer similar advantages. SQ-LNS were designed to support maternal nutrition and infant growth by providing vitamins, minerals and essential fatty acids to populations in low-resource settings^(11)^. A series of meta-analyses showed the effectiveness of SQ-LNS on health, growth and development^(12–15)^. Improved growth, particularly stature and enhanced lean body mass, can contribute to greater strength that may promote participation in PA. Previous research has shown that child stature at ages 5–17 years is positively associated with muscle strength^(16)^. Additionally, SQ-LNS is associated with improved motor skills, as a higher percentage of infants who received SQ-LNS were able to walk at 12 months old compared with control groups^(17)^. Understanding the effects of SQ-LNS on PA could provide valuable insight into the effects of early-life nutrition on child development.

We previously found that children aged 4–6 years who were exposed to SQ-LNS in early life spent more time in sedentary behaviour compared with a control group, as measured using a hip-worn accelerometer over a 1-week period^(18)^. However, there were no significant differences in MVPA between the SQ-LNS and control groups. We hypothesised that these differences could be due to fewer socioemotional difficulties in the SQ-LNS group^(18)^, such as restlessness and fidgeting associated with hyperactivity. Recent follow-up data from this trial showed that children who received SQ-LNS tended (*P* = 0·06) to have higher height-for-age z-scores at 9–11 years of age compared with children who did not receive SQ-LNS^(19)^. The difference in height-for-age z-scores was primarily among girls (SQ-LNS: 0·08 (sd 1·04); control: –0·16 (sd 1·01), *P* = 0·01), with no difference among boys (SQ-LNS: −0·16 (sd 0·85); control: –0·16 (sd 0·96), *P* = 0·97). Based on the positive relation between stature and muscle strength^(20)^, and previous meta-analyses showing improvements in motor development in children receiving SQ-LNS^(12)^, we hypothesised that adolescents in the SQ-LNS group would have higher levels of PA and lower levels of sedentary behaviour compared with those in the non-LNS group. In addition, we explored potential modifiers of SQ-LNS effects on PA.

## Methods

### Study design

#### Parent trial

The International Lipid-Based Nutrient Supplements (iLiNS) DYAD trial in Ghana was a randomised controlled trial that evaluated the efficacy of SQ-LNS given to women during pregnancy until 6 months postpartum and their children from 6 to 18 months of age. The iLiNS-DYAD-Ghana trial has been discussed in detail previously (ClinicalTrials.gov, identifier NCT00970866; https://clinicaltrials.gov/ct2/show/NCT00970866)^(21)^. Briefly, this was a partially double-blind, randomised controlled trial conducted in two semi-urban districts (Yilo and Manya Krobo) in the eastern region of Ghana, approximately 70 km north of the capital, Accra, between 2009 and 2014. The primary objectives were to evaluate the efficacy of supplementation during much of the first 1000 d on birth length and 18-month length-for-age z-scores. Pregnant women aged ≥ 18 years attending antenatal clinics in four health facilities were invited to participate in the study. Pregnant women were excluded if their antenatal card showed or they reported having had asthma, epilepsy, HIV infection, malignancy, tuberculosis or any known milk or peanut allergies. Additionally, women were also excluded if they met any of the following criteria: (i) were > 20 weeks of gestation (as determined by the antenatal clinics primarily by fundal height); (ii) were living outside the study area; (iii) were planning to move out of the study area within 2 years; (iv) were unwilling to receive fieldworkers or take study supplements; or (v) were participating in another trial.

A total of 1320 pregnant women were enrolled and randomly assigned to receive one of three interventions: (i) iron and folic acid supplement (*n* 441); until delivery and 200 mg Ca/d as placebo from delivery until 6 months postpartum; (ii) multiple micronutrient supplements until 6 months postpartum and no supplementation for their infants (*n* 439); (iii) SQ-LNS until 6 months postpartum and SQ-LNS for their infants from 6 to 18 months of age (*n* 440). The nutrient content of each supplement is shown in online Supplementary Table S1.

### Follow-up study

This sub-study was part of a larger follow-up study of the children whose mothers were enrolled in the parent iLiNS-DYAD-Ghana trial. This follow-up study was conducted from October 2022 to November 2023, when the adolescents were 11–13 years old. Adolescents were excluded from the current sub-study if their parents did not consent to their participation or if the adolescent was no longer residing in the study area (Yilo and Manya Krobo). This study was conducted according to the guidelines laid down in the Declaration of Helsinki. We obtained ethical approval for the current follow-up study from the Institutional Review Board of the University of California, Davis (IRB ID: 1489918) and the Ghana Health Service Ethics Review Committee (GHS-ERC: 027105119). After the study was explained, parents of the adolescents provided written informed consent, and adolescents provided assent by signature or thumbprint.

### Selection of participants

We targeted a minimum enrolment of 504 participants (168 per intervention arm) to participate in this sub-study assessment. For three-group comparisons, this sample size allowed us to detect a mean difference effect size of 0·37 sd or greater in the primary outcome, mean vector magnitude count per minute (VM cpm), assuming 80 % power and alpha = 0·05. We sampled children in two stages. First, we invited all 353 children who participated in the PA assessment at 4–6 years of age to participate. Next, to reach the full target sample size, a statistician not involved with data collection generated a block-randomised list of 540 additional eligible participants balanced by intervention arm, to account for attrition and participant refusals. We recruited from this list until the target sample size was achieved.

### Demographics and anthropometrics

Maternal sociodemographic information, including maternal education, was collected at baseline. Pre-pregnancy BMI was calculated from estimated pre-pregnancy weight (based on polynomial regression with gestational age, gestational age squared and gestational age cubed as predictors) and height at enrolment^(22)^. Maternal education was recorded at baseline as the number of years of formal schooling completed. The Household Food Insecurity Access Scale questionnaire was used to assess food insecurity in the household^(23,24)^. Higher values for the Household Food Insecurity Access Scale indicate higher food insecurity. As a measure of household socio-economic status (SES), a household assets index was created by including items such as radio, television, refrigerator, cell phone and stove^(25)^. Higher values in the household assets index indicate a higher SES.

Follow-up assessments were conducted when participants were 4–6 years old (*n* 1014) and again at 9–11 years (*n* 966). At both timepoints, biological (e.g. height, weight and mid-upper arm circumference) and socio-environmental (e.g. home environment, household size and household assets index) indicators were measured. At the 4–6 years follow-up^(26)^, we assessed the home environment using the Early Childhood Home Observation for the Measurement of the Environment (HOME) inventory^(27)^, which we adapted to the local context in Ghana. Our adapted version of the Early Childhood HOME contained forty-six items to measure academic stimulation, caregivers’ responsivity, desirable behaviour modelling, family lifestyle variety, language stimulation, learning materials, negative behaviour acceptance and the physical environment.

We used the Middle Childhood HOME^(28)^ to assess the home environment at the 9–11 years follow-up^(19)^; this tool consisted of fifty-eight items measuring the availability of learning materials, emotional climate, encouragement of maturity, family participation, level of active stimulation, parental involvement, parental responsivity and physical environment.

Child sex was recorded at birth. At 11–13 years, trained data collectors measured anthropometric variables according to WHO standards^(29)^. Height was measured using a stadiometer to within 0·1 cm (Seca 217). Weight was measured to the nearest 50 g with an electronic scale (Seca 875). Mid-upper arm circumference was measured around the midpoint of the upper arm to the nearest 0·1 cm with a tape measure. Measurements were taken in duplicate and in triplicate if values differed by more than 0·1 kg for weight, 0·5 cm for height or 0·5 cm for mid-upper arm circumference. Adolescent BMI z-score was calculated according to WHO growth standards^(29)^.

For the most recent follow-up at 11–13 years, we used the Early Adolescent HOME^(30)^, which contained sixty items measuring the quality and quantity of support, stimulation and structure provided to the adolescent in the home environment. Additionally, it assessed the physical and social aspects of the home environment. Adolescent school grade, student-to-teacher ratio and teacher qualifications were recorded by questionnaire. Maternal depression was assessed using the Center for Epidemiological Studies Depression Scale Revised at the adolescent 11–13 years follow-up. Pubertal development was measured using the Petersen Pubertal Developmental Scale^(31)^.

### Physical activity assessment

Accelerometers (Actigraph wGT3X-BT) were used to measure PA patterns. This method has been validated^(32)^ and has been widely used as an objective method of PA assessment among children and adults^(33–37)^. We placed an accelerometer on each participant’s non-dominant wrist to measure PA for seven consecutive days. We selected wrist-worn accelerometers due to their higher compliance rate compared with hip-worn devices^(38)^. The devices were secured to the participant’s wrist using a nylon wristband during office visits by team members. A team member initialised the accelerometer to record activity at a sample rate of 30 Hz, began recording at 05.00 h on the first day of the 7-d period and automatically stopped recording activity at 21.00 h on the 7th day. Team members instructed parents and caregivers to remove the device if it caused major discomfort or when adolescents performed water-based tasks such as laundry or bathing. The device was removed after the measurement period, and de-identified data were downloaded with Actilife software (Actilife v6.13.4; Actigraph LLC), which was then uploaded to a secure server. The primary outcome for analysis was the mean VM cpm after we removed sleep and non-wear time. The secondary outcomes were the percentage of time in sedentary behaviour, light PA and MVPA over a 7-d measuring period.

Before beginning data collection, all accelerometers underwent an initial set of quality control checks. We connected all accelerometers to the Actilife software to determine functionality, battery life, and update all device software. We conducted a small pilot study among field staff and participants aged 11–13 years who were residing in the study area but not a part of the main iLiNS-DYAD-Ghana trial. The pilot study included testing the battery life, initialisation, data collection and data download for each device (unpublished). We also used the pilot study to assess participant comfort levels of wrist-worn accelerometers, ensuring proper adherence for the 7-d measuring period (unpublished). Despite favourable pilot study results showing that all devices were functioning appropriately and there was 100 % adherence to accelerometer wear, we experienced a higher-than-expected device malfunction after data collection began in the main study, as many devices did not power on when they were returned after the 7-d data collection period. The cause of the device failures is unknown, but potential contributing factors may have included age-related wear, as the same accelerometers were used at the preschool follow-up, or greater-than-expected exposure to environmental conditions such as excessive heat and moisture. Participants with missing data due to device failure were asked to repeat the PA assessment with a different device for a second week.

After the 7-d accelerometer assessment, data collectors interviewed the adolescents about the types of activities performed in the last week using the Physical Activity Questionnaire for Older Children (PAQ-C). The PAQ-C is a ten-question form to self-report PA from the past 7 d, which we adapted to the local context^(39)^. The questionnaire was pilot tested and adapted based on socio-cultural norms, equipment availability and local naming conventions of sports or structured exercise prior to beginning data collection. For example, we removed all ice sports, and we added *Ampe*, a traditional dancing game often played by girls. All other questions remained as originally written. The questionnaire asks about specific activities, the times of day they are performed and their frequency. In addition, children were asked to report if sickness or anything else prevented them from completing normal PA.

### Physical activity data reduction

We completed all accelerometer data reduction using Actilife v6.13.4 (Actigraph LLC). We included participants in the final analysis if they had ≥ 4 total days, including at least one weekend day and two weekdays, with a minimum of 10 h of recorded activity per day. We calculated the percentage of time spent in sedentary behaviour, light PA and MVPA using cut-points previously validated for wrist-worn accelerometers for children 8–12 years old by Chandler *et al*.^(40)^. Cut points were determined using vector magnitude counts with counts from zero to 3660 classified as sedentary behaviour, 3661 to 9804 as light PA and 9805 and above as MVPA. All counts were based on 60-s epochs. Sleep period was scored based on the Sadeh *et al.* equation^(41)^, and sleep period detection was calculated using the Tudor-Locke *et al.* algorithm^(42)^. Non-wear time was defined as a minimum non-wear period of 90 consecutive minutes with zero activity counts, measured using vector magnitude. We used 30-min windows to allow for brief spikes in activity lasting up to 2 min that could be classified as part of the non-wear period. Movements lasting 2 min or less were classified as part of the 30-min period of inactivity if there was no other movement for 30 min before or after the initial movement. All sleep and non-wear time periods were excluded from the final analysis. Adolescents whose accelerometers registered 60 min or more of MVPA per day were classified as meeting the WHO minimum PA recommendations^(43)^.

All questions of the PAQ-C, except for question 10, are scored on a scale of 1–5. The 10th question does not contribute to the final score in the PAQ-C but does provide an opportunity for the children to state whether they performed their usual amount of PA during the measuring period. Composite scores on a scale of 1–5 were calculated for questions 1 and 9. For questions 2–8, we used the reported frequency, with the lowest activity reported being a 1 and the highest activity reported being a 5. We calculated the final PAQ-C summary score as the average of the scores from the nine items, excluding item 10. This resulting score, ranging from 1 to 5, indicated the child’s overall PA level for the past 7 d. A score of 1 suggests low PA, while a score of 5 implies high PA^(39)^.

### Statistical analysis

We posted a statistical analysis plan with pre-specified outcomes, potential covariates and effect modifiers on Open Science Framework (https://osf.io/bmv9d/) prior to conducting any analyses. All analyses were conducted in StataSE 17 (StataCorp LLC). We examined all continuous variables by univariate analysis to determine distribution and outliers. Outliers in mean VM cpm and percent time in sedentary behaviour were winsorised to the 1st and 99th percentile to restrict extreme values (2 % of values for each variable).

We conducted a complete case intention-to-treat analysis according to the assigned supplement group. We conducted a sensitivity analysis to examine differences between the iron and folic acid and multiple micronutrient supplement groups in the primary outcome, VM cpm. We planned to report the main results as the comparison between the SQ-LNS and combined non-LNS (iron and folic acid + multiple micronutrient supplement) groups if there were no significant differences between the iron and folic acid and multiple micronutrient supplement groups in the primary outcome. We used ANCOVA models to test the effect of SQ-LNS on continuous outcomes with statistical significance set at an alpha of 0·05. The first model was minimally adjusted, including only child sex and age at follow-up. The second model was additionally adjusted for potential covariates if they were associated with VM cpm at *P* < 0·10 in bivariate analyses. Potential covariates included maternal age, maternal education, pre-pregnancy BMI, Household Food Insecurity Access Scale, household assets index and parity. The final model was additionally adjusted for covariates assessed at follow-up if they were associated with VM cpm at *P* < 0·10. Potential covariates for the third model included the Early Childhood HOME, Middle Childhood HOME, Early Adolescent HOME, school grade, student-to-teacher ratio, teacher qualifications, maternal depression score at 10 years and child pubertal development at 10 years old.

Given that demographic and socio-economic factors may modify the effect of SQ-LNS on PA, we tested maternal education, household assets index, Household Food Insecurity Access Scale, child sex, maternal pre-pregnancy BMI, Middle Childhood HOME, Early Adolescent HOME and parity as potential modifiers of the effects of the nutrition intervention group in the statistical analysis plan. Sex differences, SES and the home environment may shape PA patterns. Those on the higher end of the SES spectrum have more resources available to support structured exercise and provide adequate nutrition; however, SQ-LNS supplementation may be more beneficial for children from lower SES backgrounds who may suffer from nutrient gaps. Additionally, children from more stimulating home environments may have more access to educational resources, high parental engagement and even a safe area to play. However, SQ-LNS may have some protective effects against lower stimulation households, as children from the iLiNS cohort who received SQ-LNS at 4–6 years and 9–11 years self-reported lower conduct problems^(44,45)^. Therefore, we assessed each of these potential effect modifiers with an interaction term in the ANCOVA model (i.e. household assets index X Group). Any statistically significant interactions at *P* < 0·10 were further examined with stratified analyses.

## Results

### Follow-up and study sample characteristics

**Fig. 1.**
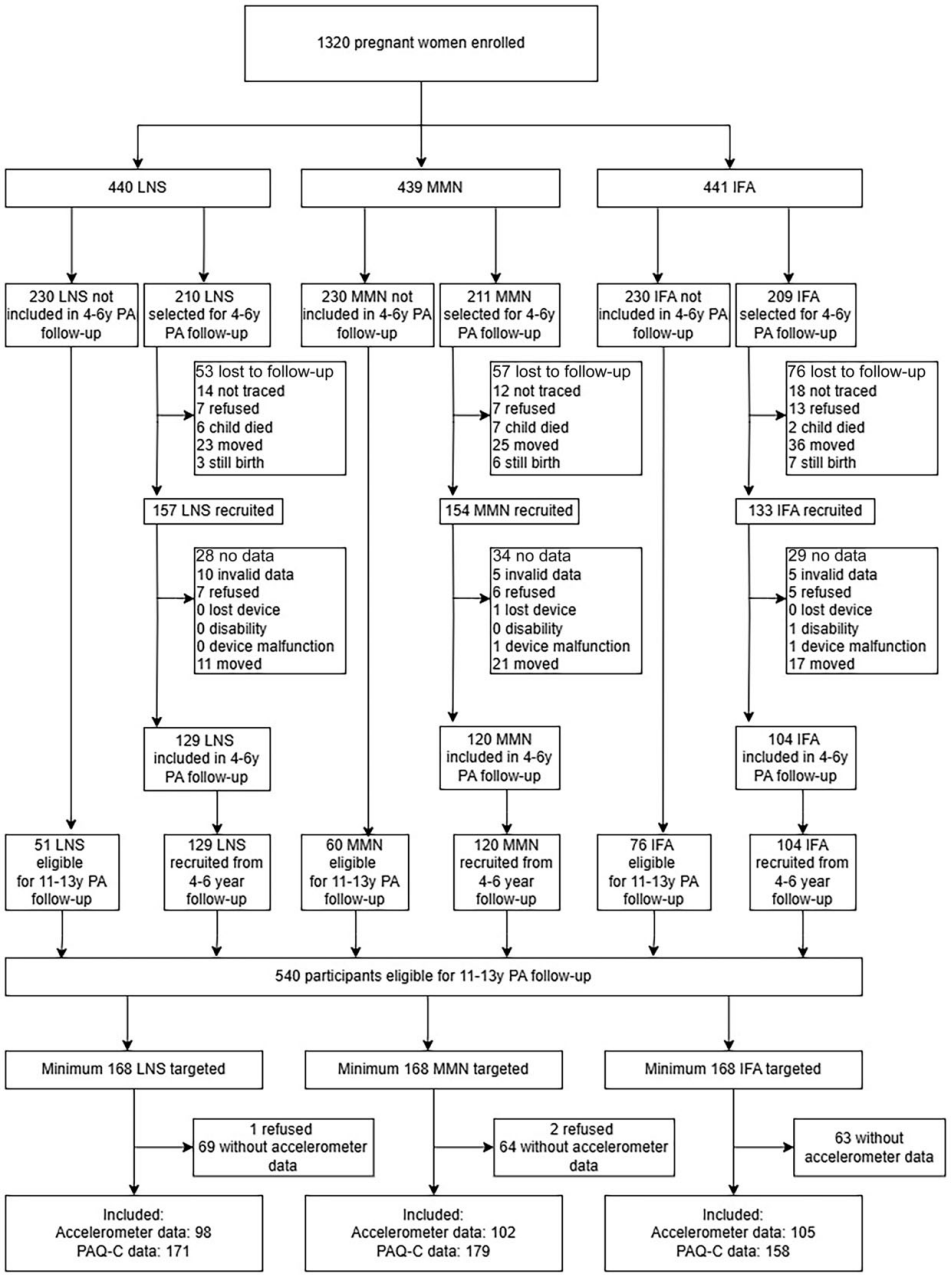
Study profile of the adolescents whose mothers were enrolled in the International Lipid-Based Nutrient Supplements trial, including reasons they were either lost to follow-up or not included in this follow-up. LNS, lipid-based nutrient supplement; MMN, multiple micronutrient supplements; IFA, iron and folic acid supplement; PA, physical activity; PAQ-C, Physical Activity Questionnaire for Older Children.

We collected accelerometer data for 305 adolescents (98 SQ-LNS and 207 non-LNS) and PAQ-C scores for 508 adolescents (171 SQ-LNS and 337 non-LNS) (Fig. 1). Three caregivers (*n* 1 SQ-LNS; *n* 2 multiple micronutrient supplement) did not give parental consent to participate in this sub-study. By the end of the sub-study, 24 out of the initial 28 accelerometers were no longer functioning, which prevented data collection from 199 of the originally targeted participants. Background characteristics were similar among participants with accelerometer and PAQ-C data (Tables 1–2). There were no differences in background characteristics between the SQ-LNS and non-LNS groups. Similarly, the adolescents did not differ in anthropometric measurements at follow-up between the two groups. The participants included in the accelerometry analysis had older mothers, were more likely to be female and were less likely to have nulliparous mothers compared with the cohort not included (online Supplementary Table S2). Children with PAQ-C data were generally similar to those not included in the analysis, except they had older mothers, were less likely to have nulliparous mothers, were more likely to be female and spent fewer hours in school (online Supplementary Table S3).

**Table 1.** Background characteristics of participants by intervention group at baseline and follow-up with accelerometer data^*^

	SQ-LNS		Non-LNS	
	(*n* 98)		(*n* 207)	
	Mean	sd	Mean	sd
Maternal characteristics at baseline				
Age (years)	28·5	5·6	27·2	5·1
Formal education (years)	7·8	3·8	7·2	3·5
Pre-pregnancy BMI^†^ (kg/m^2^)	24·8	3·9	24·5	4·8
Household asset index^‡^	−0·08	1·1	0·07	1·0
Household food insecurity access score	2·3	4·0	3·0	4·8
Nulliparous (%)	25·6		25·5	
Child characteristics				
Age at 11–13 years (years)	11·9	0·4	11·8	0·4
Weight at 11–13 years (kg)	37·5	7·6	36·7	8·3
Height at 11–13 years (cm)	148·4	7·9	146·8	7·9
BMIZ at 11–13 years (years)	17·0	2·8	16·9	2·7
MUAC at 11–13 years (cm)	20·7	2·8	20·5	2·7
Sex-assigned-at-birth (% male)	38·8		44·0	
Season at measurement^§^ (% dry)	31·6		30·3	
Mean hours in school	7·8	0·6	7·8	0·5
Valid days of accelerometer wear (days)	7·0	0·1	7·0	0·2

BMIZ, BMI z-score; MUAC, mid-upper arm circumference; SQ-LNS, small-quantity lipid-based nutrient supplement.

Results based on *t* test or *χ* ^2^.

* Values are mean (sd) or % (*n*/total). The non-LNS group refers to iron and folic acid and multiple micronutrient groups.

† Estimated pre-pregnancy BMI was calculated from height at enrolment and estimated pre-pregnancy weight (based on polynomial regression with gestation age, gestational age squared and gestational age cubed as predictors).

‡ Household asset score is a proxy for household socio-economic status and was constructed based on household ownership of a set of assets, and principal component analysis was used to create an index (mean of zero and sd of one). A higher value represents a higher socio-economic status.

§ Wet and dry seasons were classified based on the bi-modal rainy season. The rainy season was classified as April through July and September to November. The dry season was classified as December to March.

**Table 2.** Background characteristics of participants by intervention group at baseline and follow-up with PAQ-C data^*^

	SQ-LNS		Non-LNS	
	(*n* 171)		(*n* 337)	
	Mean	sd	Mean	sd
Maternal characteristics at baseline				
Age (years)	27·5	5·5	27·1	5·2
Formal education (years)	7·9	3·7	7·5	3·3
Pre-pregnancy BMI^†^ (kg/m^2^)	24·9	4·7	24·5	4·7
Household asset index^‡^	−0·05	1·0	0·08	1·0
Household food insecurity access score	2·0	3·6	2·7	4·4
Nulliparous (%)	28·7		28·2	
Child characteristics				
Age at 11–13 years (years)	11·8	0·4	11·8	0·4
Weight at 11–13 years (kg)	37·2	8·2	36·1	8·0
Height at 11–13 years (cm)	148·1	8·0	146·5	7·9
BMIZ at 11–13 years (years)	16·8	2·8	16·7	2·5
MUAC at 11–13 years (cm)	20·5	2·9	20·2	2·7
Sex-assigned-at-birth (% male)	44·4		46·0	
Season at measurement^§^ (% dry)	31·6		31·6	
Mean hours in school	7·8	0·6	7·8	0·5

BMIZ, BMI z-score; MUAC, mid-upper arm circumference; SQ-LNS, small-quantity lipid-based nutrient supplement; PAQ-C, Physical Activity Questionnaire for Older Children.

Results based on *t* test or *χ* ^2^.

* Values are mean (sd) or % (*n*/total). The non-LNS group refers to iron and folic acid (IFA) and multiple micronutrient (MMN) groups.

† Estimated pre-pregnancy BMI was calculated from height at enrolment and estimated pre-pregnancy weight (based on polynomial regression with gestation age, gestational age squared and gestational age cubed as predictors).

‡ Household asset score is a proxy for household socio-economic status and was constructed based on household ownership of a set of assets, and principal component analysis was used to create an index (mean of zero and sd of one). A higher value represents a higher socio-economic status.

§ Wet and dry seasons were classified based on the bi-modal rainy season. The rainy season was classified as April through July and September to November. The dry season was classified as December to March.

### Description of current physical activity levels

Among those with accelerometry data, the average wear time was 7 d and did not differ between groups (*P* = 0·75). Overall, most of their waking hours were spent in sedentary behaviour (65·8 % of the time), followed by light PA (29·2 % of the time), and lastly, MVPA (4·9 % of the time, Table 3). Males were, on average, more active, spending more time in MVPA (5·9 % of the time) than females (4·2 %) and less time in sedentary behaviour (64·8 % of the time) than females (66·6 %). Only 50 % of adolescents with accelerometer data met the recommended minimum of 60 min of MVPA daily, including 64·2 % of males and 39·1 % of females.

**Table 3.** Descriptive accelerometer data and PAQ-C scores of the overall sample, stratified by sex, among all participants with accelerometer data or PAQ-C scores

Variable	*n*	Mean/(%)	sd	*P* ^*^
Percentage of time in:				
Sedentary		65·8	5·6	
Male	129	64·8	5·5	0·005
Female	176	66·6	5·6	
Light PA		29·2	4·6	
Male	129	29·3	4·4	0·80
Female	176	29·1	4·7	
MVPA		4·9	2·3	
Male	129	5·9	2·5	< 0·001
Female	176	4·2	1·9	
Average proportion of children with ≥ 60 min MVPA/d (%)		50·0		
Male	129	64·2		< 0·001
Female	176	39·1		
PAQ-C scores		2·6	0·7	
Male	231	2·8	0·7	< 0·001
Female	277	2·4	0·7	

MVPA, moderate-to-vigorous physical activity; PAQ-C, Physical Activity Questionnaire for Older Children; PA, physical activity.

* Results based on *t* test for continuous variables and *χ* ^2^ tests for categorical variables.

### Physical Activity Questionnaire for Older Children scores and responses

Males averaged significantly higher PAQ-C scores compared with females (mean (sd); male: 2·76 (sd 0·7); female: 2·45 (sd 0·7); *P* < 0·001, Table 3). Several activities showed high participation rates. This suggests that these activities are more popular or more accessible to adolescents in this study area. Most notably, 85·9 % of females and 90·5 % of males (overall 88 %) reported walking to school at least once in the past 7 d (online Supplementary Table S4). Additionally, 76·2 % of adolescents reported that they engaged in jogging or running at least once in the previous 7 d. Football (soccer) was common among males (87·4 %) but infrequent among females (28·2 %). Some activities showed low-to-moderate engagement, with 18·2 % of adolescents engaging in hopscotch, 36·4 % jumping rope or skipping and 46·3 % playing *Ampe* at least once in the 7-d measuring period. Many activities had low participation, that is, 0·8–3·5 % of adolescents engaged in swimming, tennis, skateboarding or rollerblading and basketball. Like football, a few activities showed large differences by sex. *Ampe* was common among females (72·2 %) but uncommon among males (15·2 %). Similarly, females had greater participation in dancing (74 %) than males (59·7 %). Finally, there was a noticeable difference in bicycling by sex, as more males (48·5 %) engaged in cycling than females (12·2 %).

Sixty percent of adolescents reported participating in PA during physical education classes in school. During recess, 23·8 % of adolescents reported sedentary behaviour like sitting down, talking, reading or doing schoolwork. The majority (50·5 %) reported low-to-moderate PA, reporting that they stood, walked or ran around a little bit. However, a small percentage (4·8 %) reported high PA levels during recess. PA during lunch was similar to PA during recess, with a small percentage of adolescents engaging in high PA. Over a quarter of all adolescents surveyed indicated no PA after school or in the evenings. After school, the adolescents have similar levels of PA as recess and lunch, with only a small percentage of adolescents being physically active. Over 80 % of adolescents indicated that they were at least a little active on the weekends, with a small percentage being very physically active. Overall, 90·6 % of adolescents reported that they had engaged in their normal amounts of PA during the measurement period.

### Effect of small-quantity lipid-based nutrient supplements on physical activity outcomes

We did not find any significant differences between the iron and folic acid and multiple micronutrient supplement groups in the primary outcome, VM cpm; therefore, we combined the iron and folic acid and multiple micronutrient supplement groups to serve as the control group. There were no significant differences between the SQ-LNS and non-LNS groups in any of the pre-specified outcomes (VM cpm, PAQ-C scores and percentage of time in sedentary behaviour, light PA, MVPA) (Table 4). Results were similar in models minimally adjusted for sex and when adjusting for additional covariates.

**Table 4.** Effect of SQ-LNS on physical activity and sedentary behaviour

Variable	*n*	SQ-LNS		Non-LNS		Model 1	Model 2	Model 3
		Mean	sd	Mean	sd	*P* ^*^	*P* ^†^	*P* ^‡^
Accelerometry								
Mean VM cpm	305	2885	457	2977	497	0·16	0·33	0·34
Percentage of time in:								
Sedentary	305	66·7	5·5	65·4	5·6	0·10	0·19	0·18
Light	305	28·5	4·7	29·5	4·5	0·12	0·18	0·11
MVPA	305	4·7	2·0	5·0	2·4	0·26	0·47	0·82
PAQ-C								
Mean score	507	2·5	0·7	2·6	0·7	0·12	0·18	0·21

The control group is iron and folic acid and multiple micronutrient groups combined. Results based on ANCOVA.

VM cpm, vector magnitude counts per minute; MVPA, moderate-to-vigorous physical activity; PAQ-C, Physical Activity Questionnaire for Older Children; SQ-LNS, small-quantity lipid-based nutrient supplement.

* Model 1 was adjusted for child age and sex at follow-up only.

† Model 2 was additionally adjusted for child sex-assigned-at-birth, age at follow-up, maternal age at enrolment, maternal education, pre-pregnancy BMI, household food insecurity access scale, household asset index and parity.

‡ Model 3 was additionally adjusted for the following factors collected at follow-up: Early adolescent home observation for measurement of the environment, maternal depression score at 10 years, child pubertal development at 10 years old and current grade in school.

### Interaction of small-quantity lipid-based nutrient supplements with effect modifiers

Among the forty-five interaction tests (nine effect modifiers by five outcomes) conducted, four (8·9 %) interactions were statistically significant at *P* < 0·1. This is consistent with what we would expect to be significant by chance. Therefore, the results of the interaction tests are not presented.

## Discussion

In this follow-up study, we aimed to describe current PA patterns of youths with mothers who had enrolled in a prenatal nutrition intervention study and to examine the long-term effects of pre- and postnatal SQ-LNS on adolescent PA and sedentary behaviour. Overall, approximately 50 % of adolescents did not meet the recommended minimum of 60 min of MVPA daily. There were large differences in PA between males and females, with males being more active and more likely to meet PA recommendations. There was no significant effect of SQ-LNS on VM cpm, percentage of time in sedentary behaviour, light PA, MVPA or PAQ-C scores.

Limited research exists examining the long-term effects of early-life interventions on adolescent PA. In a previous study from this cohort conducted when children were 4–6 years old, we found significant differences in PA, as the SQ-LNS group had lower VM cpm and spent more time in sedentary behaviour than the non-LNS group^(18)^. The difference in PA levels was previously attributed to differences in social-emotional development and behavioural problems, wherein the children exposed to SQ-LNS had fewer behavioural problems compared with those in the non-LNS group^(45)^. However, this positive effect of SQ-LNS on social-emotional problems was not sustained into early adolescence^(44)^, which could explain the differing PA results seen in this later follow-up. Our results contrast with other evaluations showing that nutritional supplementation led to higher early childhood PA levels^(7,8)^. However, those studies were conducted at much younger ages, 8 months old to 4 years old, and investigators did not explore the sustained effects of nutritional supplementation on PA patterns into adolescence. Therefore, the effects of early-life nutritional supplementation on PA likely attenuate over time.

Studies documenting adolescent PA levels in Ghana are limited, but these levels seem to be improving after a steep decline in the last decade^(1,46–49)^. Despite the improvement of overall PA seen as recently as the 2018 Report Card on Physical Activity, the most concerning pattern is the low amount of PA performed by females, as males are reportedly more active, consistent with PA patterns globally^(1,47,49)^. Although this most recent report is promising compared with global inactivity, PA levels have not been examined recently. The adolescents we assessed appear more active than adolescents worldwide, with 50 % not meeting PA recommendations compared with 80 % in global estimates^(1)^. This may be due to various reasons, such as walking to school and having more opportunities for free play. We found that 88 % of adolescents reported walking to school at least once during the week of PA assessment. Additionally, 88 % of adolescents reported running or jogging at least once in the past week, which can be another reason this cohort had higher levels of PA compared with global estimates.

We found that, similar to global estimates, males appear more active than females, with an average of 64·2 % of males and 39·1 % of females meeting the minimum 60 min of MVPA daily. Even though 90·5 % of males and 85·9 % of females reported walking to school at least one time in the previous week, this alone is insufficient to enable the adolescents to meet the PA recommendations, either because the duration was short or the intensity was too low to have registered as MVPA on the accelerometers. Typically, females in Ghana tend to hold more domestic responsibilities than males and might be required to help with household chores, prepping the family meal or caring for younger children in the home after school. In contrast, males may have more free time during this period, which may allow males to participate in more structured daily movements or sports. For example, 87 % of males indicated that they participated in football or ball sports during the week of PA measurement, whereas only 28 % of females reported this. Even though girls tend to have more domestic responsibility, 72 % played *Ampe*, a high-energy game involving dancing, clapping and jumping, at least 1–2 times in the past week, but the duration of time spent in this activity was shorter than would be needed to achieve the minimum time spent in MVPA.

Strengths of our study include the randomised design of the early-life intervention and blinding of the PA and sedentary behaviour outcomes among intervention groups to assessors. Further, we collected self-reported PA using the PAQ-C, which we used to determine the activities with higher engagement among adolescents during the PA measuring period. All accelerometers were initialised using standard operating procedures, and PA data were scored using cut-offs previously shown to accurately classify sedentary behaviour and PA using wrist-worn accelerometers. Finally, we conducted a small pilot study to ensure adherence to accelerometer wear among the study population, which resulted in favourable results.

Limitations of this study include the high rate of missing data due to non-functional devices, which resulted in us not being able to measure the PA of 199 participants. However, with our final sample size of 305 participants and a two-group comparison, we still had 80 % power to detect a relatively small effect size of ≥ 0·20 sd. Another limitation is that accelerometers cannot record activity types. To address this, we administered the PAQ-C to participants to describe PA behaviour and activities in the past week. However, the PAQ-C does not explicitly ask about sedentary behaviour. Future work should interview adolescents about both physical and sedentary activities to get a more thorough view of activities performed during PA assessment. Although these results provide valuable insight into adolescent PA patterns in Ghana, there is limited generalisability within this cohort as adolescents with accelerometer data differed in their mothers’ age, parity and the sex of the child compared with those not included in this study.

Contrary to our hypothesis, PA levels did not differ between early adolescents exposed to SQ-LNS *in utero* from 18 months of age compared with children in the control groups. This suggests that early-life nutrition supplements may not be a major contributor to PA at this age. We found that males are more active and more likely to meet PA recommendations than females. Due to the importance of PA in adolescence, further research should be conducted to examine the factors that influence PA levels in urban and rural areas, particularly among females, to help develop policies to improve PA patterns.

## Supporting information

Fajardo et al. supplementary material Fajardo et al. supplementary material

## Supplementary material

For supplementary material/s referred to in this article, please visit https://doi.org/10.1017/S0007114525105126.
