# Supplementary material for: The effects of small-quantity lipid-based nutrient supplements on adolescent physical activity and sedentary behaviour: a follow-up of the International Lipid-Based Nutrient Supplements-DYAD-Ghana trial
Source: Br J Nutr. 2025 Sep 19;134(6):508–17. doi: 10.1017/S0007114525105126 (PMC12623127; doi:10.1017/S0007114525105126)
Supplement: Fajardo et al. supplementary material [file S0007114525105126sup001.docx]

Supplementary material for manuscript titled: The effects of small-quantity lipid-based nutrient supplements on adolescent physical activity and sedentary behavior: a follow-up of the iLiNS-DYAD Ghana trial, by Jonnatan Fajardo

**Supplemental Table S1**. Nutrient and energy contents of supplements used in the iLiNS trial in Ghana^1^

|  | **IFA** | **MMN** | **Maternal SQ-LNS** | **Child SQ-LNS** |
| --- | --- | --- | --- | --- |
| Ration per day | 1 capsule | 1 capsule | 20-g sachet | 20-g sachet |
| Total energy, kcal | 0 | 0 | 118 | 118 |
| Protein, g | 0 | 0 | 2.6 | 2.6 |
| Fat, g | 0 | 0 | 10 | 9.6 |
| Linoleic acid, g | 0 | 0 | 4.59 | 4.46 |
| α-Linolenic acid, g | 0 | 0 | 0.59 | 0.58 |
| Vitamin A, μg RE | 0 | 800 | 800 | 400 |
| Vitamin C, mg | 0 | 100 | 100 | 30 |
| Vitamin B-1, mg | 0 | 2.8 | 2.8 | 0.3 |
| Vitamin B-2, mg | 0 | 2.8 | 2.8 | 0.4 |
| Niacin, mg | 0 | 36 | 36 | 4 |
| Folic acid, μg | 400 | 400 | 400 | 80 |
| Pantothenic acid, mg | 0 | 7 | 7 | 1.8 |
| Vitamin B-6, mg | 0 | 3.8 | 3.8 | 0.3 |
| Vitamin B-12, μg | 0 | 5.2 | 5.2 | 0.5 |
| Vitamin D, mg | 0 | 10 | 10 | 5 |
| Vitamin E, mg | 0 | 20 | 20 | 6 |
| Vitamin K, μg | 0 | 45 | 45 | 30 |
| Iron, mg | 60 | 20 | 20 | 6 |
| Zinc, mg | 0 | 30 | 30 | 8 |
| Copper, mg | 0 | 4 | 4 | 0.34 |
| Calcium, mg | 0 | 0 | 280 | 280 |
| Phosphorus, mg | 0 | 0 | 190 | 190 |
| Potassium, mg | 0 | 0 | 200 | 200 |
| Magnesium, mg | 0 | 0 | 65 | 40 |
| Selenium, μg | 0 | 130 | 130 | 20 |
| Iodine, μg | 0 | 250 | 250 | 90 |
| Manganese, mg | 0 | 2.6 | 2.6 | 1.2 |

^1^IFA, iron and folic acid capsule; MMN, multiple micronutrient supplement capsule; SQ-LNS, small quantity lipid-based nutrient supplement. Information from table previously published ^(1)^.

**Supplemental Table S2** Background characteristics of participants at baseline and follow-up that were included and excluded from the follow-up study with accelerometer data^1^

|  | **Included** | | **Excluded^2^** | |  |
| --- | --- | --- | --- | --- | --- |
|  | **(n=305)** | | **(n = 1015)** | |  |
|  | Mean | SD | Mean | SD | p-value |
| Maternal characteristics at baseline |  |  |  |  |  |
| Age (y) | 27.6 | 5.2 | 26.4 | 5.6 | <0.001 |
| Formal education (y) | 7.4 | 3.6 | 7.7 | 3.7 | 0.15 |
| Pre-pregnancy BMI^3^ (kg/m^2^) | 24.6 | 4.5 | 24.5 | 4.3 | 0.80 |
| Household asset index^4^ | 0.02 | 1.0 | -0.00 | 1.0 | 0.69 |
| Household food insecurity access score | 2.8 | 4.5 | 2.6 | 4.2 | 0.38 |
| Nulliparous (%) | 25.6 |  | 36.3 |  | <0.001 |
| Child characteristics |  |  |  |  |  |
| Age at 11-13y (y) | 11.8 | 0.4 | 11.8 | 0.5 | 0.85 |
| Weight at 11-13y (kg) | 37.0 | 8.1 | 36.5 | 8.2 | 0.46 |
| Height at 11-13y (cm) | 147.3 | 7.9 | 147.5 | 8.0 | 0.76 |
| BMIZ at 11-13y (y) | 16.9 | 2.7 | 16.6 | 2.7 | 0.17 |
| MUAC at 11-13y (cm) | 20.6 | 2.7 | 20.2 | 2.8 | 0.05 |
| Sex (% male) | 42.3 |  | 51.6 |  | 0.005 |
| Mean hours in school | 7.8 | 0.5 | 7.8 | 0.6 | 0.08 |
|  |  |  |  |  |  |

BMI, body mass index; BMIZ, BMI z-score; MUAC, mid-upper arm circumference; SQ-LNS, small-quantity lipid-based nutrient supplement; PAQ-C, Physical Activity Questionnaire for Older Children.

Results based on t-test or chi-square
^1^ Values are mean (SD) or % [n/total].

^2^ Children were excluded due to insufficient accelerometers (n=196), parents did not consent (n=3), not selected for study (n=813)

^3^ Estimated pre-pregnancy BMI was calculated from height at enrolment and estimated pre-pregnancy weight (based on polynomial regression with gestation age, gestational age squared, and gestational age cubed as predictors).

^4^ Household asset score is a proxy for household socioeconomic status and was constructed based on household ownership of a set of assets and principal component analysis was used to create an index (mean of zero and standard deviation of one). A higher value represents a higher socioeconomic status.

**Supplemental Table S3** Background characteristics of participants at baseline and follow-up that were included and excluded from the follow-up study with PAQ-C data^1^

|  | **Included** | | **Excluded^2^** | |  |
| --- | --- | --- | --- | --- | --- |
|  | **(n=508)** | | **(n = 812)** | |  |
|  | Mean | SD | Mean | SD | p-value |
| Maternal characteristics at baseline |  |  |  |  |  |
| Age (y) | 27.2 | 5.3 | 26.4 | 5.6 | 0.011 |
| Formal education (y) | 7.6 | 3.5 | 7.6 | 3.8 | 0.94 |
| Pre-pregnancy BMI^3^ (kg/m^2^) | 24.7 | 4.7 | 24.4 | 4.1 | 0.35 |
| Household asset index^4^ | 0.04 | 1.0 | -0.02 | 1.0 | 0.35 |
| Household food insecurity access score | 2.5 | 4.2 | 2.7 | 4.3 | 0.29 |
| Nulliparous (%) | 28.3 |  | 37.2 |  | <0.001 |
| Child characteristics |  |  |  |  |  |
| Age at 11-13y (y) | 11.8 | 0.4 | 11.9 | 0.5 | <0.001 |
| Weight at 11-13y (kg) | 36.5 | 8.1 | 36.9 | 8.3 | 0.42 |
| Height at 11-13y (cm) | 147.0 | 8.0 | 147.9 | 8.0 | 0.10 |
| BMIZ at 11-13y (y) | 16.7 | 2.6 | 16.7 | 2.8 | 0.95 |
| MUAC at 11-13y (cm) | 20.3 | 2.7 | 20.3 | 2.8 | 0.79 |
| Sex (% male) | 45.5 |  | 52.0 |  | 0.02 |
| Mean hours in school | 7.8 | 0.5 | 7.9 | 0.6 | 0.03 |
|  |  |  |  |  |  |

BMI, body mass index; BMIZ, BMI z-score; MUAC, mid-upper arm circumference; SQ-LNS, small-quantity lipid-based nutrient supplement; PAQ-C, Physical Activity Questionnaire for Older Children.

Results based on t-test or chi-square
^1^ Values are mean (SD) or % [n/total].

^2^ Children were excluded due to parents not giving consent (n=3), not selected for study (n=809)

^3^ Estimated pre-pregnancy BMI was calculated from height at enrolment and estimated pre-pregnancy weight (based on polynomial regression with gestation age, gestational age squared, and gestational age cubed as predictors).

^4^ Household asset score is a proxy for household socioeconomic status and was constructed based on household ownership of a set of assets and principal component analysis was used to create an index (mean of zero and standard deviation of one). A higher value represents a higher socioeconomic status.

**Supplemental Table S4**. Descriptive frequency of PAQ-C^1^ responses stratified by sex and total responses

|  |  | Female  n=277 | Male  n=231 | p-value |
| --- | --- | --- | --- | --- |
| PAQ-C Score | | 2.45 (0.7) | 2.76 (0.7) | <0.001 |
|  |  |  |  |  |
| Physical activity in your spare time: Have you done any of the following activities in the past 7 days (last week)? | | | | |
|  | | Female n (%) | Male n (%) | Total n (%) |
| Hopscotch | |  |  |  |
|  | No | 216 (78.0%) | 196 (84.8%) | 412 (81.1%) |
|  | 1-2 | 49 (17.7%) | 25 (10.8%) | 74 (14.6%) |
|  | 3-4 | 11 (4.0%) | 8 (3.5%) | 19 (3.7%) |
|  | 5-6 | 0 (0.0%) | 2 (0.9%) | 2 (0.4%) |
|  | 7 times or more | 1 (0.4%) | 0 (0.0%) | 1 (0.2%) |
| Jump rope & skipping | |  |  |  |
|  | No | 171 (61.7%) | 152 (65.8%) | 323 (63.6%) |
|  | 1-2 | 68 (24.5%) | 60 (26.0%) | 128 (25.2%) |
|  | 3-4 | 32 (11.6%) | 14 (6.1%) | 46 (9.1%) |
|  | 5-6 | 4 (1.4%) | 3 (1.3%) | 7 (1.4%) |
|  | 7 times or more | 2 (0.7%) | 2 (0.9%) | 4 (0.8%) |
| Walking to school | |  |  |  |
|  | No | 39 (14.1%) | 22 (9.5%) | 61 (12.0%) |
|  | 1-2 | 17 (6.2%) | 10 (4.3%) | 27 (5.3%) |
|  | 3-4 | 24 (8.7%) | 20 (8.7%) | 44 (8.7%) |
|  | 5-6 | 195 (70.7%) | 177 (76.6%) | 372 (73.4%) |
|  | 7 times or more | 1 (0.4%) | 2 (0.9%) | 3 (0.6%) |
| Walking for exercise | | | | |
|  | No | 219 (79.1%) | 163 (70.6%) | 382 (75.2%) |
|  | 1-2 | 40 (14.4%) | 46 (19.9%) | 86 (16.9%) |
|  | 3-4 | 10 (3.6%) | 14 (6.1%) | 24 (4.7%) |
|  | 5-6 | 8 (2.9%) | 5 (2.2%) | 13 (2.6%) |
|  | 7 times or more | 0 (0.0%) | 3 (1.3%) | 3 (0.6%) |
| Bicycling | |  |  |  |
|  | No | 243 (87.7%) | 119 (51.5%) | 362 (71.3%) |
|  | 1-2 | 28 (10.1%) | 67 (29.0%) | 95 (18.7%) |
|  | 3-4 | 4 (1.4%) | 31 (13.4%) | 35 (6.9%) |
|  | 5-6 | 0 (0.0%) | 6 (2.6%) | 6 (1.2%) |
|  | 7 times or more | 2 (0.7%) | 8 (3.5%) | 10 (2.0%) |
| Jogging or running | |  |  |  |
|  | No | 69 (24.9%) | 52 (22.5%) | 121 (23.8%) |
|  | 1-2 | 70 (25.3%) | 47 (20.3%) | 117 (23.0%) |
|  | 3-4 | 82 (29.6%) | 71 (30.7%) | 153 (30.1%) |
|  | 5-6 | 44 (15.9%) | 44 (19.0%) | 88 (17.3%) |
|  | 7 times or more | 12 (4.3%) | 17 (7.4%) | 29 (5.7%) |
| Aerobics (high knees, squats) | |  |  |  |
|  | No | 255 (92.1%) | 201 (87.0%) | 456 (89.8%) |
|  | 1-2 | 14 (5.1%) | 25 (10.8%) | 39 (7.7%) |
|  | 3-4 | 3 (1.1%) | 4 (1.7%) | 7 (1.4%) |
|  | 5-6 | 5 (1.8%) | 1 (0.4%) | 6 (1.2%) |
|  | 7 times or more | No Response | No Response | No Response |
| Swimming | |  |  |  |
|  | No | 273 (98.6%) | 224 (97.0%) | 497 (97.8%) |
|  | 1-2 | 4 (1.4%) | 6 (2.6%) | 10 (2.0%) |
|  | 3-4 | 0 (0.0%) | 1 (0.4%) | 1 (0.2%) |
|  | 5-6 | No Response | No Response | No Response |
|  | 7 times or more | No Response | No Response | No Response |
| Tennis | |  |  |  |
|  | No | 272 (98.2%) | 218 (94.4%) | 490 (96.5%) |
|  | 1-2 | 3 (1.1%) | 9 (3.9%) | 12 (2.4%) |
|  | 3-4 | 1 (0.4%) | 1 (0.4%) | 2 (0.4%) |
|  | 5-6 | 1 (0.4%) | 3 (1.3%) | 4 (0.8%) |
|  | 7 times or more |  |  |  |
| Dancing | |  |  |  |
|  | No | 72 (26.0%) | 93 (40.3%) | 165 (32.5%) |
|  | 1-2 | 114 (41.2%) | 92 (39.8%) | 206 (40.6%) |
|  | 3-4 | 68 (24.5%) | 31 (13.4%) | 99 (19.5%) |
|  | 5-6 | 19 (6.9%) | 12 (5.2%) | 31 (6.1%) |
|  | 7 times or more | 4 (1.4%) | 3 (1.3%) | 7 (1.4%) |
| Skateboard or rollerblading | |  |  |  |
|  | No | 277 (100.0%) | 227 (98.3%) | 504 (99.2%) |
|  | 1-2 | 0 (0.0%) | 4 (1.7%) | 4 (0.8%) |
|  | 3-4 | No Response | No Response | No Response |
|  | 5-6 | No Response | No Response | No Response |
|  | 7 times or more | No Response | No Response | No Response |
| Soccer (football, ball) | |  |  |  |
|  | No | 199 (71.8%) | 29 (12.6%) | 228 (44.9%) |
|  | 1-2 | 60 (21.7%) | 62 (26.8%) | 122 (24.0%) |
|  | 3-4 | 18 (6.5%) | 72 (31.2%) | 90 (17.7%) |
|  | 5-6 | 0 (0.0%) | 39 (16.9%) | 39 (7.7%) |
|  | 7 times or more | 0 (0.0%) | 29 (12.6%) | 29 (5.7%) |
| Volleyball | |  |  |  |
|  | No | 264 (95.3%) | 222 (96.1%) | 486 (95.7%) |
|  | 1-2 | 9 (3.2%) | 6 (2.6%) | 15 (3.0%) |
|  | 3-4 | 2 (0.7%) | 3 (1.3%) | 5 (1.0%) |
|  | 5-6 | 2 (0.7%) | 0 (0.0%) | 2 (0.4%) |
|  | 7 times or more | No Response | No Response | No Response |
| Basketball | |  |  |  |
|  | No | 273 (98.6%) | 221 (96.1%) | 494 (97.4%) |
|  | 1-2 | 3 (1.1%) | 9 (3.9%) | 12 (2.4%) |
|  | 3-4 | 1 (0.4%) | 0 (0.0%) | 1 (0.2%) |
|  | 5-6 | No Response | No Response | No Response |
|  | 7 times or more | No Response | No Response | No Response |
| Ampe | |  |  |  |
|  | No | 77 (27.8%) | 196 (84.8%) | 273 (53.7%) |
|  | 1-2 | 90 (32.5%) | 27 (11.7%) | 117 (23.0%) |
|  | 3-4 | 70 (25.3%) | 6 (2.6%) | 76 (15.0%) |
|  | 5-6 | 31 (11.2%) | 1 (0.4%) | 32 (6.3%) |
|  | 7 times or more | 9 (3.2%) | 1 (0.4%) | 10 (2.0%) |
| Hide and seek or tag | |  |  |  |
|  | No | 212 (76.5%) | 165 (71.4%) | 377 (74.2%) |
|  | 1-2 | 34 (12.3%) | 31 (13.4%) | 65 (12.8%) |
|  | 3-4 | 21 (7.6%) | 20 (8.7%) | 41 (8.1%) |
|  | 5-6 | 9 (3.2%) | 15 (6.5%) | 24 (4.7%) |
|  | 7 times or more | 1 (0.4%) | 0 (0.0%) | 1 (0.2%) |

PAQ-C, Physical Activity Questionnaire for Older Children

Results based on t-test^1^ We adapted the PAQ-C to the local context based on socio-cultural norms, equipment availability, and local naming conventions of sports or structured exercise.
